# Influence of tree cover on carcass detection and consumption by facultative vertebrate scavengers

**DOI:** 10.1002/ece3.10935

**Published:** 2024-04-01

**Authors:** Elke Wenting, Patrick A. Jansen, Luke Pattipeilohy, Peter van Lunteren, Henk Siepel, Frank van Langevelde

**Affiliations:** ^1^ Department of Environmental Sciences Wageningen University and Research Wageningen The Netherlands; ^2^ Department of Animal Ecology and Physiology Radboud Institute for Biological and Environmental Sciences, Radboud University Nijmegen The Netherlands; ^3^ Smithsonian Tropical Research Institute Panama City Panama; ^4^ Addax Data Science Utrecht The Netherlands

**Keywords:** carcass decomposition, carrion, facultative scavengers, tree cover, wild boar

## Abstract

Scavenging mammals and vultures can exploit and deplete carcasses much faster than other birds and invertebrates. Vultures are strongly influenced by habitat type, e.g. tree cover, since they rely on their eyesight to detect carcasses. It remains unclear whether and how facultative scavengers – both other birds and mammals – are influenced by tree cover and how that affect carcass decomposition time, which in turn affects biodiversity and ecological processes, including the cycle of energy and nutrients. We studied whether the carcass detection and consumption, hence carcass decomposition speed, by facultative avian and mammalian scavengers varies with tree cover in areas without vultures. Fresh mammal carcasses were placed in different landscapes across the Netherlands at locations that widely varied in tree cover. Camera traps were used to record carcass exploitation by facultative avian and mammalian scavengers and to estimate carcass decomposition time. We found that carcass detection and consumption by birds, wild boar, and other mammals varied between locations. Carcass decomposition speed indeed increased with carcass detection and exploitation by mammals, especially by wild boar. However, this variation was not related to tree cover. We conclude that tree cover is not a major determinant of carcass exploitation by facultative scavengers in areas without obligate scavengers and large carnivores.

## Graphical abstract

We studied whether the carcass detection and consumption, hence carcass decomposition speed, by facultative avian and mammalian scavengers varies with tree cover in areas without vultures. Carcass decomposition speed indeed increased with carcass detection and exploitation by mammals, especially by wild boar. However, this variation was not related to tree cover.

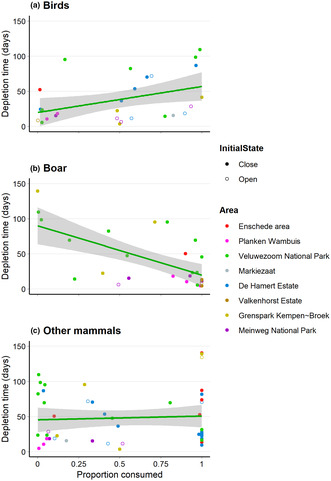

## 1 INTRODUCTION

The decomposition of dead animal bodies – carcasses – has potentially far‐reaching effects on biodiversity and ecological processes, including the cycle of energy and nutrients (Moore et al., 2004; Swift et al., 1979) – a key natural process for ecosystem functioning (Ngai & Srivastava, 2006). Several studies have shown that carcasses can alter local nutrient dynamics (e.g. Barton et al., 2019; Macdonald et al., 2014; Quaggiotto et al., 2019). In addition, carcass availability in general subsidizes facultative scavengers and can be important for species survival under harsh conditions, e.g. during winter (e.g. Wilson & Wolkovich, 2011).

The species assemblages involved in the carcass decomposition process can vary enormously, with a wide spectrum of possible assemblages (e.g. Olson et al., 2016; Wenting et al., 2022). At the one extreme, carcasses are entirely decomposed by invertebrate scavengers and microbial decomposers (Bump et al., 2009; Carter et al., 2007). At the other extreme, carcasses are almost exclusively consumed by vertebrate scavengers – i.e. birds and mammals (DeVault et al., 2003). The first extreme would proceed slower, providing a longer time window for scavenger species to arrive, hence strongly promote biodiversity (e.g. Barton & Evans, 2017; Blazquez et al., 2009; Wenting et al., 2022). The second extreme would strongly promote the nutrient cycle by faster redistributing carcass‐derived nutrients over large areas via scavengers (e.g. Bump et al., 2009; Parmenter & MacMahon, 2009; Wenting et al., 2023). Most carcasses, however, decompose through an in‐between scenario resulting in highly variable decomposition speeds (Olson et al., 2016; Wenting et al., 2022).

The carcass decomposition process is influenced by many biotic and abiotic factors, including ambient temperature (e.g. Parmenter & MacMahon, 2009; Selva et al., 2005), carcass type (Olson et al., 2016), carcass size (e.g. Moleón et al., 2015; Ogada et al., 2012; Turner et al., 2017), and local species assemblage (e.g. Farwig et al., 2014; Wenting et al., 2022). Also, habitat type may be relevant as, for example, Arrondo et al. (2019) found that carcasses located in open areas were detected and consumed earlier compared to carcasses located in more heterogeneous and forested areas. Similarly, Pardo‐Barquín et al. (2019) found that the amount of tree cover lowered scavenger richness and diversity, particularly hindering avian scavengers to access carcasses. The amount of tree cover, therefore, is expected to be an important driving factor for scavengers in detecting and consuming carcasses, hence affecting carcass depletion speed.

However, studies focussing on the effects of habitat type are predominantly biased towards invertebrate scavengers (e.g. Barton & Evans, 2017; Farwig et al., 2014). Moreover, understanding the effect of habitat type on the carcass decomposition process is biased towards areas where vultures are present (e.g. Byrne et al., 2019; Gavashelishvili & McGrady, 2006; Hill et al., 2018; Houston, 1986; Oliva‐Vidal et al., 2022). Since vultures are obligate scavengers and are known to have an enormous effect on carcass removal from ecosystems (e.g. Cortés‐Avizanda et al., 2014; Mateo‐Tomás et al., 2017; Ogada et al., 2012; Sebastián‐González et al., 2021), these findings might not necessarily apply to areas where vultures are absent. Facultative scavengers are the most prominent consumers of carcasses in areas without vultures (DeVault et al., 2003), but the importance of habitat type on facultative avian and mammalian scavengers in areas without vultures remains unclear.

Facultative avian and mammalian scavengers differ in their adaptations and abilities to detect and consume carcasses (e.g. Selva et al., 2005; Wenting et al., 2022). In general, avian scavengers seem to be better adapted to carcass detection in open areas as they are mainly guided by their eyesight (e.g. Selva et al., 2005; Wilmers et al., 2003), while carcass detection of mammalian scavengers is mainly driven by olfactory cues (e.g. Ruxton & Houston, 2004; Selva et al., 2005; Stahler et al., 2002). Also, as a result of their morphology, the average maximum intake rate of mammals is generally larger compared to birds (Van Gils et al., 2007). Consequently, carcasses that are dominantly exploited by mammals – wild boar (*Sus scrofa*, henceforth ‘boar’) in particular (Wenting et al., 2022) – would faster decompose than when birds are dominantly present.

Thus, whether avian or mammalian scavengers dominate the carcass decomposition process matters because it may greatly affect the decomposition speed. We expect that the carcass decomposition process is strongly influenced by the amount of tree cover. We tested five predictions: (1) the denser the tree cover, the longer it takes before carcasses are first detected and first scavenged by birds, but the faster carcasses are first detected and first scavenged by boars or other mammals; (2) the denser the tree cover, the lower the proportion of carcass consumed by birds, but the higher the proportion consumed by boars or other mammals; (3) the sooner carcasses are first detected or first scavenged by birds, boars or other mammals, the higher the proportion of carcass consumed by these groups; (4) the carcass decomposition speed is not influenced by time to first detection or first scavenging by birds, but is accelerated by time to first detection or first scavenging by boars or other mammals; and (5) the carcass decomposition speed is not influenced by the proportion of carcass consumed by birds, but is accelerated when the proportions consumed by boars or other mammals increase.

## 2 METHODS

### 2.1 Study area

**FIGURE 1:**
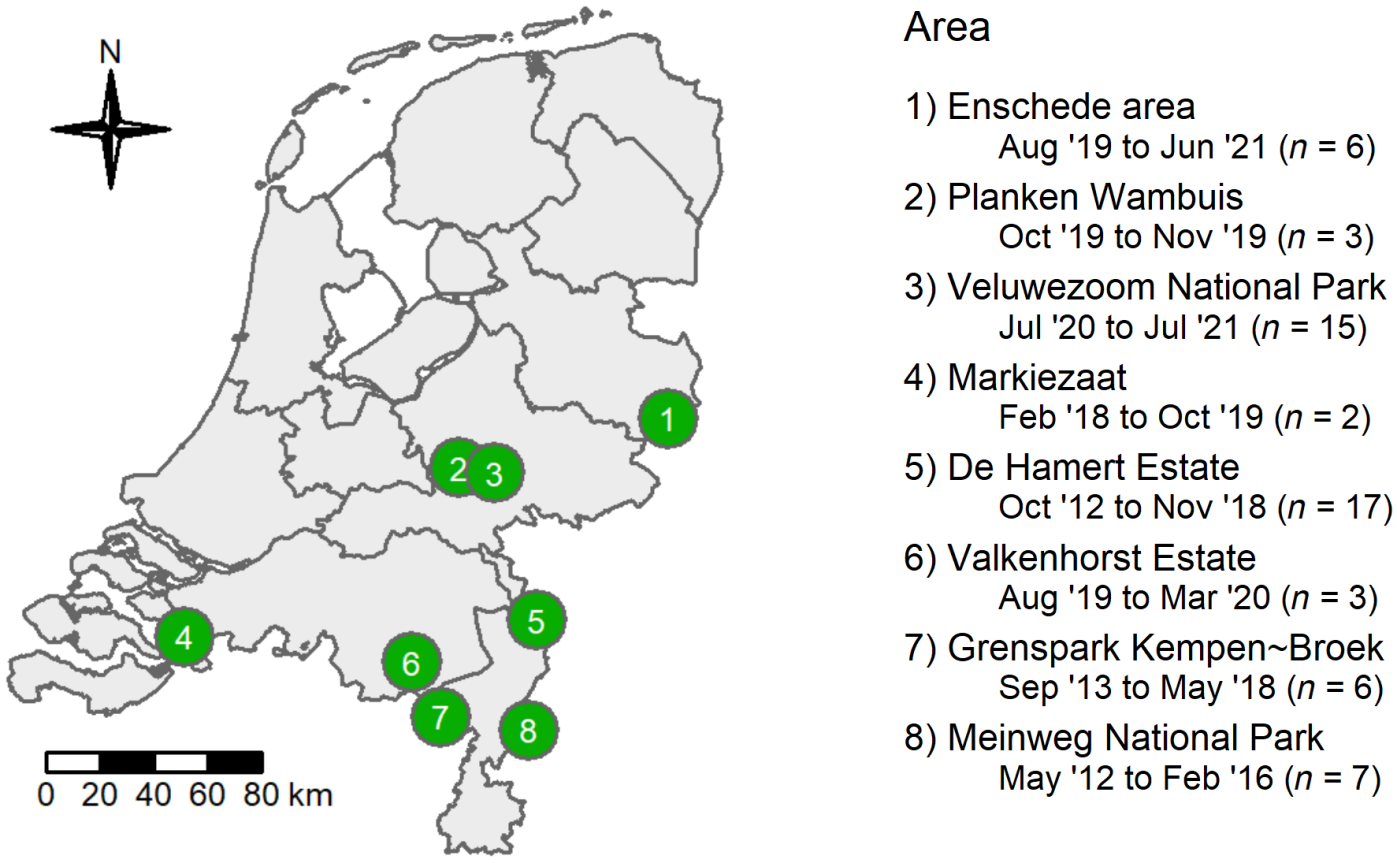
Map of the Netherlands showing the areas where we monitored carcasses until depletion. The period of monitoring and the number of monitored carcasses per area are indicated.

We monitored the vertebrate animals that visited 59 carcasses in eight Dutch protected areas, in different periods between May 2012 and July 2021 (Figure 1). We selected locations within these areas that represented the variation of tree cover, e.g. heathlands with barely any tree cover, forest edges with intermediate tree cover, and dense forest with high tree cover. Some areas occupied all gradations of tree cover, e.g. Veluwezoom National Park and Meinweg National Park. Other areas occupied different gradations as, for instance, Planken Wambuis only occupied higher tree cover, while Valkenhorst Estate only occupied intermediate tree cover. The forest types were similar among the areas, consisting of a mix of deciduous and coniferous forest.

The majority of the monitored carcasses – 49 out of 59 – were also used to analyse the effect of functional differences in scavenger communities on the carcass decomposition speed (Wenting et al., 2022). Not all areas inhabited the same species (Table S1.1); hence, we included area as random factor in the statistical analyses. Most noteworthy, avian and mammalian scavengers were present in all areas, while boar was absent in Markiezaat and De Hamert Estate but present in the other areas. In all study areas, the species with the highest capacity to consume carcasses – large carnivores and vultures (Sebastián‐González et al., 2021) – were absent at the time of monitoring.

### 2.2 Field methods

We used the same protocol as described in Wenting et al. (2022), using motion‐triggered infrared camera traps – all part of the Bushnell TrophyCam product line – to monitor fresh carcasses until depletion. We attached the camera traps to trees, shrubs or actively placed poles at a distance of 2 m from the carcass, about 1 m height and slightly bent forward pointing towards the ground, depending on the local circumstances. All carcasses were positioned with the abdomen or back to the camera, and tied at the front and rear legs to trees or poles using natural ropes to prevent dragging out of view. The camera traps were set to videos of 60 s per trigger, with a two‐ or three‐second interval – depending on the exact camera model – between the triggers. We visited the carcasses every 2 weeks on average to replace the 32 or 64 GB SD card and to renew the batteries. We minimized the time spent and the number of persons present at the carcass site as much as possible to reduce possible anthropogenic disturbance.

We only included carcasses of which we monitored the whole decomposition process in the analyses, resulting in a total of 59 carcasses. Only entire carcasses were monitored, i.e. no guts only. The carcasses were obtained from roadkills, except for Planken Wambuis and Veluwezoom National Park, where we obtained the carcasses from regular culling. No animals were killed for the purpose of this study. In total, we monitored carcasses of 34 roe deer (*Capreolus capreolus*), 3 red deer (*Cervus elaphus*), 7 fallow deer (*Dama dama*), 4 European badgers (*Meles meles*), one domestic sheep (*Ovis orientalis aries*), and 10 boars. For practical reasons, the carcass placement was on a non‐randomized stratified case‐by‐case basis. The stratification was based on the variation in tree cover among and within the areas, to ensure that we monitored carcasses over the entire gradient of tree cover present in the areas. We included carcass species as random factor in the statistical analyses since not all carcass species might attract the same scavenger species (e.g. Butler‐Valverde et al., 2022). For instance, carnivore carcasses in particular – European badger in our case – might attract other scavenger species than herbivore carcasses (Moleón et al., 2017). However, the scavenger communities of the carcasses used in our study did not differ between carcasses or areas (Wenting et al., 2022).

### 2.3 Annotation camera trapping videos

The collected videos were uploaded to the online platform Agouti (Casaer et al., 2019), from which the footage was annotated, as described in Wenting et al. (2022). For this study specifically, we annotated per video (1) the species that visited the carcass; (2) the number of individuals on each video; (3) whether direct scavenging behaviour, i.e. eating or collecting carcass materials, was shown; and (4) the scavenger group to which the species belonged. We focused on the three scavenger groups, as defined by Wenting et al. (2022), that were most relevant for our study: (1) Birds; (2) Mammals, and (3) Wild boar. The group of birds consisted of common raven (*Corvus corax*), common buzzard (*Buteo buteo*) and carrion crow (*Corvus corone*). The group Mammals (henceforth ‘other mammals’), consisted of beech marten (*Martes foina*), domestic cat (*Felis catus*), domestic dog (*Canis lupus familiaris*), European polecat (*Mustela putorius*) and red fox (*Vulpes vulpes*). The birds' group was characterized by a prevalence for carcasses in the active stage of decomposition, while the other mammals' group was present in all stages of decomposition. The other mammals' group was characterized by their overall larger body size compared to the birds. The third group, wild boar, was characterized by large body size, high percentage of eating behaviour, and high amount of intraspecific behaviour, indicating larger group sizes and more social behaviour compared to any of the other groups (Wenting et al., 2022).

We expanded the carcasses monitored in Wenting et al. (2022) with 10 additional carcasses. Some of these 10 carcasses were visited by species that were not yet included in the scavenger groups defined by Wenting et al. (2022). We assessed these species based on the same criteria of, e.g., at least 30 observations and showing direct scavenging behaviour. We noticed that we had to add three scavenger species to the scavenger groups: European badger, pine marten (*Martes martes*) and European robin (*Erithacus rubecula*). Based on their behaviour towards the carcasses and their prevalence, we classified European badger and pine marten as other mammals. European robin was classified as occasional scavenger (Wenting et al., 2022) and hence not relevant for the purpose of our study.

Per carcass, we noted (1) the time to carcass depletion, based on the date of carcass placement and the date of depletion; (2) the percentage of tree cover (see below); (3) the mean daily temperature, based on the nearest weather station (KNMI, 2021); (4) the area the carcass was located; (5) the carcass species; (6) the start month calculated from the first monitored carcass, enabling us to correct for temporal autocorrelation; and (7) carcass initial state, i.e. whether the skin was such severely damaged due to the cause of death that we considered it opened at the moment of carcass placement. Carcasses were considered as fully decomposed when none of the carcass remains were visible anymore.

Furthermore, we calculated per carcass (1) the time until first detection and (2) first scavenging event per scavenger group; and (3) the proportion of carcass consumption per scavenger group. The proportion of carcass consumption per scavenger group was based on the total number of observations per carcass, thus calculated as the proportion of consuming scavengers per group. The calculated proportions were rescaled to avoid zeros and ones in the analyses (Smithson & Verkuilen, 2006).

### 2.4 Tree cover calculation

We retroactively calculated the percentage of tree cover (henceforth ‘tree cover’) for the monitored carcasses. We loaded the shapefiles in Google Earth, where we used aerial photos to calculate the tree cover. This calculation was based on 100 random sampling points using a 30 m radius circle around each carcass location, of which we created shapefiles with the sf package (Pebesma, 2018). The 30 m corresponds with approximately 0.3 ha. This was chosen to reflect the properties of the location of the carcass as well as the properties of the landscape immediately around it, accounting for the sightlines of overflying birds from different angles (Ilangakoon et al., 2015). We divided the year into four periods to take into account the annual change of leaf area index in temperate forests: (1) the leaf production period in May; (2) the leaf constant period from June through September; (3) the leaf senescent period from October through November; and (4) the leaf dormant period from December through April (Blackburn & Milton, 1995; Croft et al., 2014; Gond et al., 1999; Wang et al., 2005). We based the photographic analysis on the time of the year a carcass was monitored. However, due to infrequent adequate satellite photos, it was not possible to calculate the tree cover for all sites in the production and senescent period. In those cases, we calculated the average tree cover of the constant and dormant period. We always ensured that the vegetation type did not change between the used aerial photo and the monitoring period.

### 2.5 Statistical analyses

We used mixed‐effects Cox models (Therneau & Therneau, 2015) to analyse how the time till first detection or first scavenging depended on tree cover. We tested this per scavenger group, with ambient temperature and carcass initial state as covariates, and area, carcass species, and start month as random factors. For boar, we excluded the carcasses monitored in Markiezaat and De Hamert Estate since this species did not occur in these areas.

We used Beta‐distributed generalized linear mixed models with a logit link (Brooks et al., 2023) to analyse the proportion of carcass consumed per scavenger group. We tested whether tree cover was related to the proportion consumed with three models – one for each group – with the proportion consumed as dependent variable, the percentage of tree cover as independent variable, ambient temperature and carcass initial state as covariates, and area, carcass species, and start month as random factors. We tested whether the time to first detected or first scavenged was related to the proportion consumed with two models per group (six in total), with the proportion consumed as dependent variable, the time to first detection or first scavenging as independent variable, ambient temperature and carcass initial state as covariates, and area, carcass species, and start month as random factors. We only included the carcasses that were visited by the corresponding scavenger group in the analyses.

We used linear mixed‐effects models (Kuznetsova et al., 2017) to analyse the carcass decomposition speed. We used two models per scavenger group (six in total) to test whether the time until first detection or first scavenging was related to the depletion time, with depletion time as dependent variable, time to first detection or first scavenging as independent variable, ambient temperature and carcass initial state as covariates, and area, carcass species, and start month as random factors. We analysed whether the carcass decomposition speed was influenced by proportion of carcass consumed with three models – one per scavenger group – with depletion time as dependent variable, proportion of carcass consumed as independent variable, ambient temperature and carcass initial state as covariates, and area, carcass species, and start month as random factors. Again, we only included the carcasses that were visited by the corresponding scavenger group in the analyses.

All statistical analyses were done in R version 4.3.1 (R Core Team, 2023). See Table S2 for an overview of all test statistics.

## 3 RESULTS

The camera traps recorded a total of 13,122 videos of vertebrates visiting the 59 carcasses that we monitored, of which 11,570 videos belonged to the scavenger groups Birds, Other mammals and Wild boar, that we included in the analyses. Direct scavenger behaviour – i.e. eating or collecting carcass materials (Wenting et al., 2022) – was annotated in 9488 of these videos. After multiplying with the number of individuals counted per video, there were 15,142 observations of direct scavenging behaviour, that we used to calculate the proportion of carcass consumption per scavenger group. One of the carcasses, monitored at Veluwezoom National Park, was visited by occasional scavengers only and was therefore excluded from further analyses.

The monitored carcasses were placed under tree cover varying from 0 to 99 per cent. The time till first detection and first scavenging varied from less than a day to 43 days, and the depletion time varied from 3.5 to 140 days.

### 3.1 First detection and first scavenging

**FIGURE 2:**
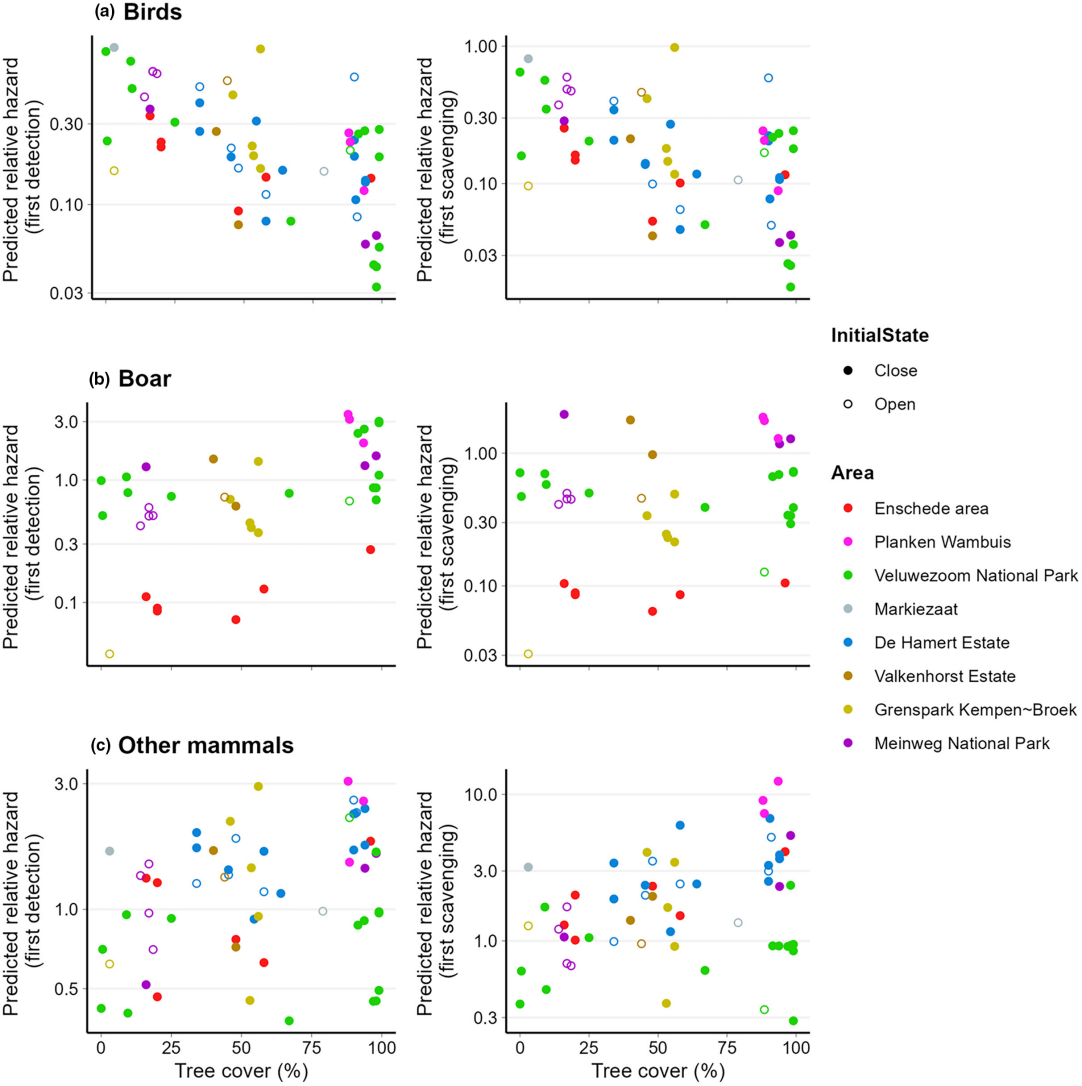
Predicted relative hazard of being first detection or first scavenging event of birds (a), boar (b), or other mammals (c) versus the percentage of tree cover.

We found that tree cover did not explain time of first carcass detection by any of the scavenger groups (Figure 2a–c), neither for birds (*β* = −0.837, SE = 0.584, *p* = .150), boars (*β* = 1.292, SE = 0.663, *p* = .52), nor other mammals (*β* = 0.913, SE = 0.560, *p* = .100). The same applied to time to first scavenging (Figure 2a–c; birds: *β* = −0.684, SE = 0.619, *p* = .270; boar: *β* = 0.126, SE = 0.644, *p* = .840; other mammals: *β* = 0.866, SE = 0.601, *p* = .150). We found, however, that increasing mean daily temperature reduced the predicted relative hazard of first detection (*β* = −0.117, SE = 0.044, *p* = .007) and first scavenging (*β* = −0.153, SE = 0.051, *p* = .003) by birds, implying that carcasses were later detected with increasing ambient temperature. Initially opened carcasses had a lower predicted relative hazard of first scavenged by boar compared to initially closed carcasses, implying that it took longer before opened carcasses were scavenged for the first time by boars compared to initially closed carcasses (*β* = −1.555, SE = 0.776, *p* = .045).

### 3.2 Carcass consumption

**FIGURE 3:**
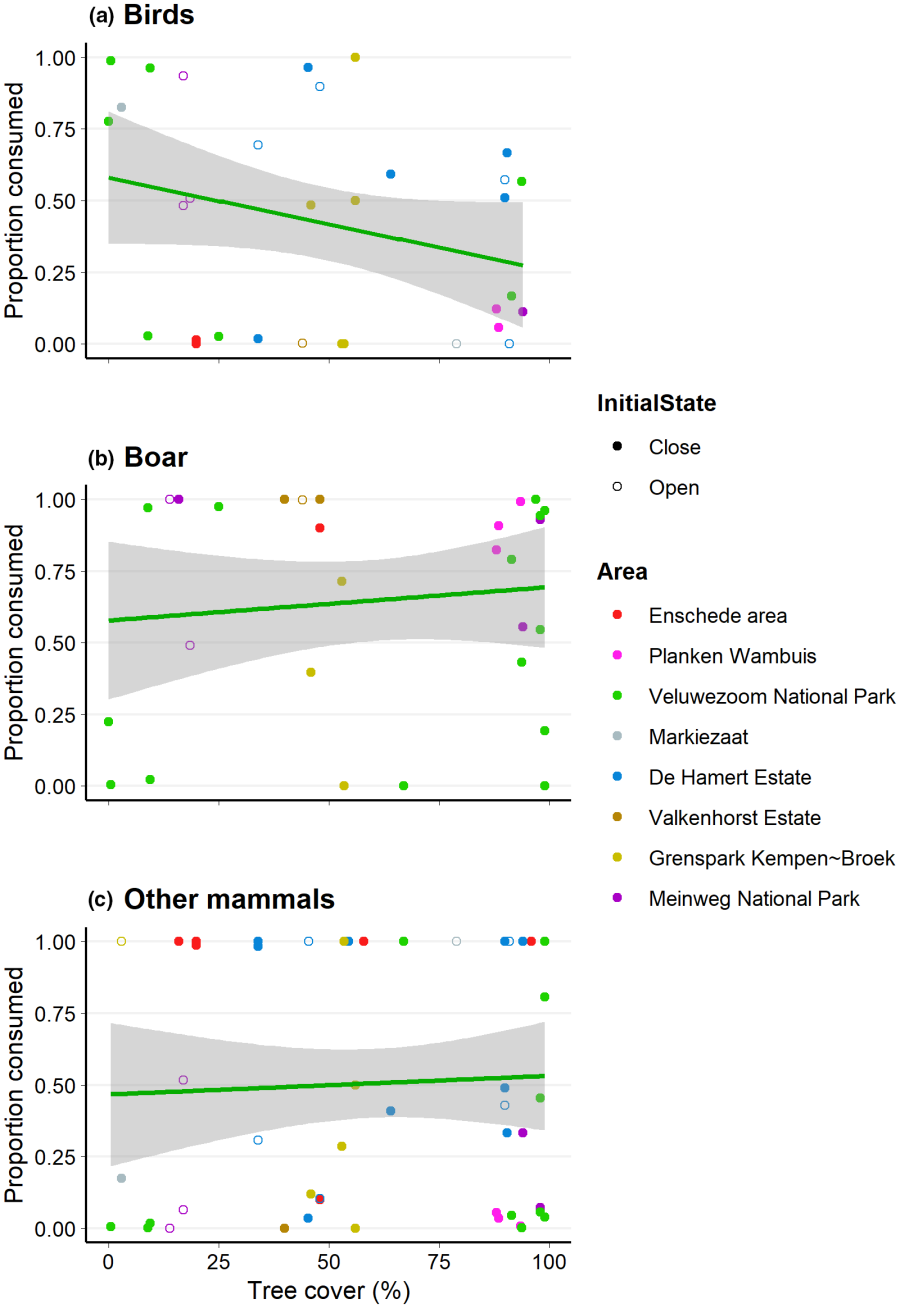
The proportion of carcass consumption attributed to birds (a), boar (b), or other mammals (c) versus the percentage of tree cover.

Tree cover was not related to the proportion of carcass consumed by birds (Figure 3a; *β* = −0.889, SE = 0.952, *p* = .350), boar (Figure 3b; *β* = −0.379, SE = 0.846, *p* = .654), or other mammals (Figure 3c; *β* = 0.337, SE = 0.637, *p* = .597). Also, none of the covariates – mean daily temperature (birds: *β* = −0.012, SE = 0.090, *p* = .895; boar: *β* = 0.008, SE = 0.057, *p* = .887; other mammals: *β* = 0.062, SE = 0.041, *p* = .130) and carcass initial state (birds: *β* = −0.147, SE = 0.658, *p* = .823; boar: *β* = 0.437, SE = 0.995, *p* = .661; other mammals: *β* = 0.154, SE = 0.501, *p* = .758) – was significant for any scavenger group.

**FIGURE 4:**
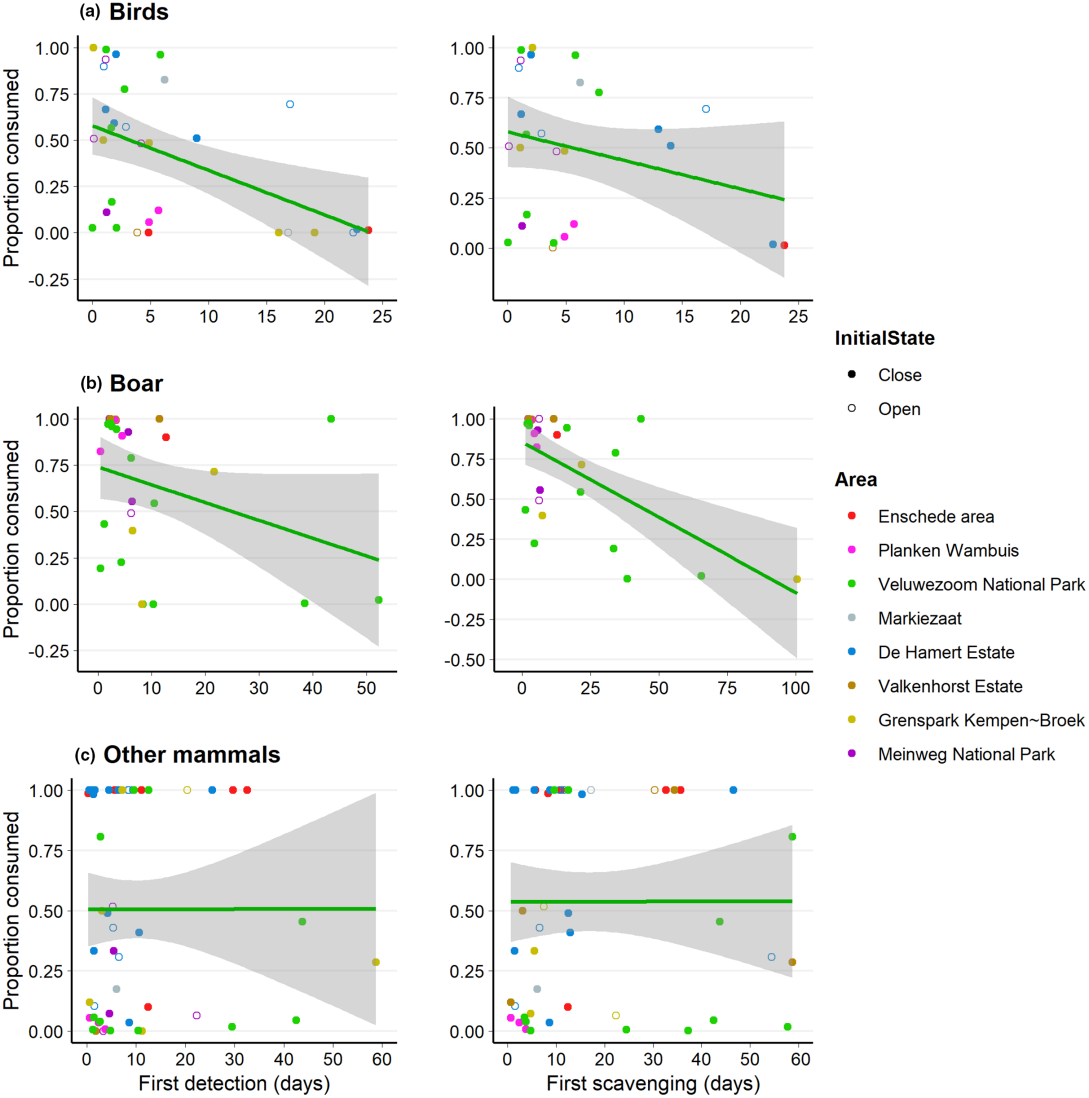
The proportion of carcass consumption attributed to birds (a), boar (b), or other mammals (c) versus the days until the first detection or first scavenging event.

The proportion of carcass consumed by birds was higher when birds detected the carcasses sooner (Figure 4a; *β* = −0.125, SE = 0.037, *p* < .001). Boar consumed a larger proportion when they sooner scavenged a carcass for the first time (Figure 4b; *β* = −0.038, SE = 0.012, *p* = .001). The proportion of carcass consumed by other mammals was not influenced by the time of first detection or first scavenging (Figure 4c; *β* = 0.008, SE = 0.016, *p* = .625; *β* = 0.002, SE = 0.011, *p* = .840, respectively). Also, neither for days to first detection nor first scavenging, any of the covariates – mean daily temperate (birds: *β* = 0.005, SE = 0.066, *p* = .938; *β* = 0.059, SE = 0.071, *p* = .412, respectively; boar: *β* = −0.006, SE = 0.054, *p* = .910; *β* = 0.093, SE = 0.053, *p* = .080, respectively; other mammals: *β* = 0.066, SE = 0.044, *p* = .100; *β* = 0.073, SE = 0.044, *p* = .100, respectively) or carcass initial state (*β* = 0.027, SE = 0.569, *p* = .962; *β* = −0.159, SE = 0.668, *p* = .812, respectively; boar: *β* = 0.433, SE = 1.011, *p* = .668; *β* = 0.874, SE = 0.789, *p* = .268, respectively; other mammals: *β* = 0.075, SE = 0.475, *p* = .874; *β* = −0.106, SE = 0.516, *p* = .837, respectively) – was significant.

### 3.3 Depletion time

**FIGURE 5:**
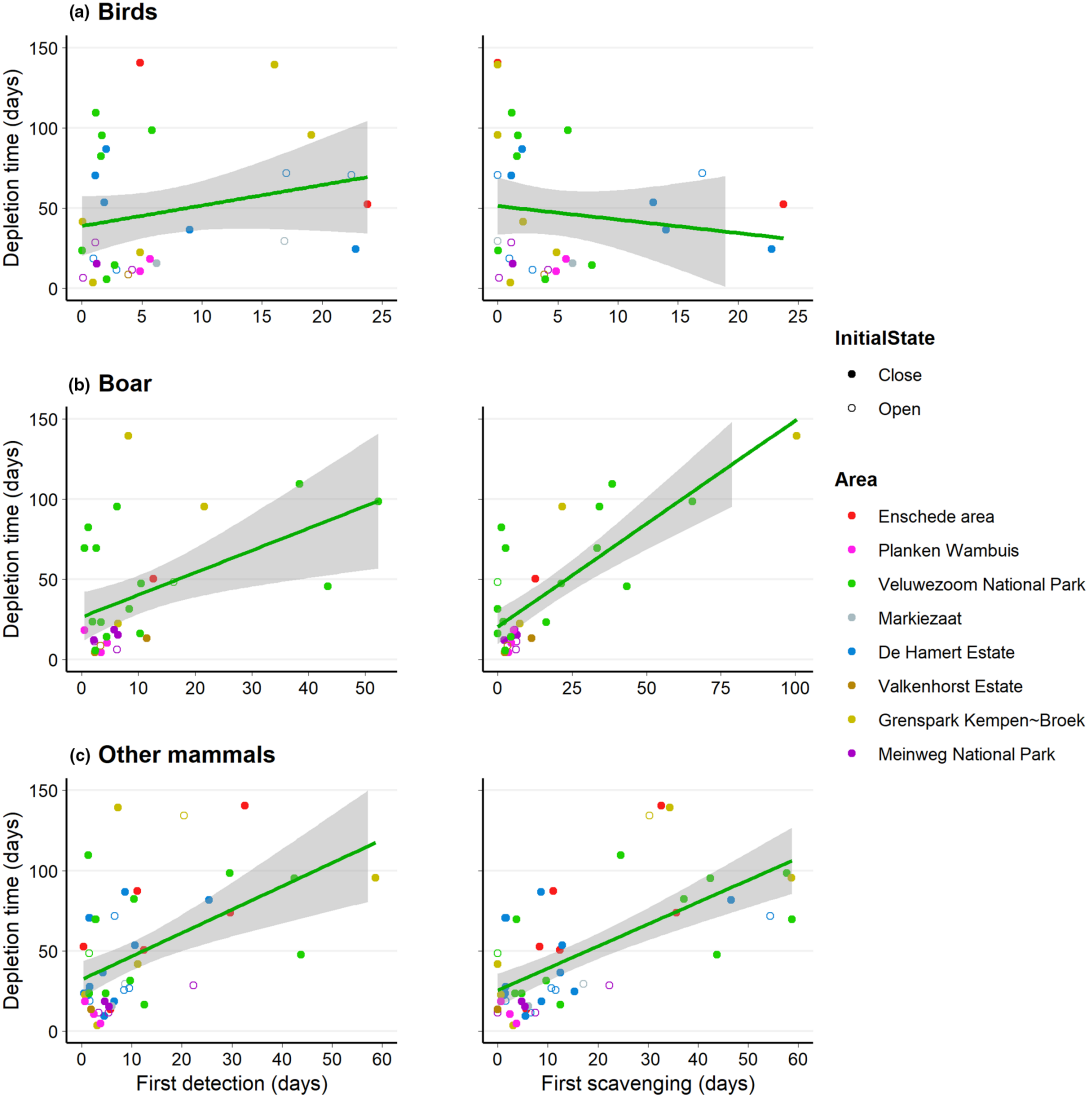
The days until first detection or first scavenging event of birds (a), boar (b), or other mammals (c) versus the days until carcass depletion.

The time to first detection or first scavenging by birds was not related to the carcass depletion time (Figure 5a; Table S2.4). We found that carcasses faster decomposed when they were sooner first detected (*β* = 1.130, SE = 0.409, df = 12.975, *p* = .016) or first scavenged (*β* = 1.230, SE = 0.190, df = 23.867, *p* < .001) by boars (Figure 5b). We also found that carcasses faster decomposed when they were sooner first detected (*β* = 1.231, SE = 0.364, df = 45.649, *p* = .002) or first scavenged (*β* = 1.304, SE = 0.228, df = 44.993, *p* < .001) by other mammals (Figure 5c). Mean daily temperature was not, neither for first detection (birds: *β* = 2.814, SE = 1.558, df = 21.378, *p* = .085; boar: *β* = −0.478, SE = 1.325, df = 13.114, *p* = .724; other mammals: *β* = 0.716, SE = 0.944, df = 36.434, *p* = .453) nor first scavenging (birds: *β* = 3.089. SE = 1.542, df = 19.716, *p* = .059; boar: *β* = −1.444, SE = 0.952, df = 25.421, *p* = .142; other mammals: *β* = 1.174, SE = 0.780, df = 33.161, *p* = .151), related to the time to depletion. The same applied to carcass initial state, which was not, nor for first detection (birds: *β* = −27.709, SE = 14.706, df = 26.118, *p* = .071; boar: *β* = −8.307, SE = 17.753, df = 24.324, *p* = .644; other mammals: *β* = −0.869, SE = 11.016, df = 46.855, *p* = .938) nor first scavenging (birds: *β* = −27.946, SE = 14.981, df = 24.716, *p* = .074; boar: *β* = −11.289, SE = 13.843, df = 25.945, *p* = .422; other mammals: *β* = −3.036, SE = 9.433, df = 46.822, *p* = .749), related to the time to depletion.

**FIGURE 6:**
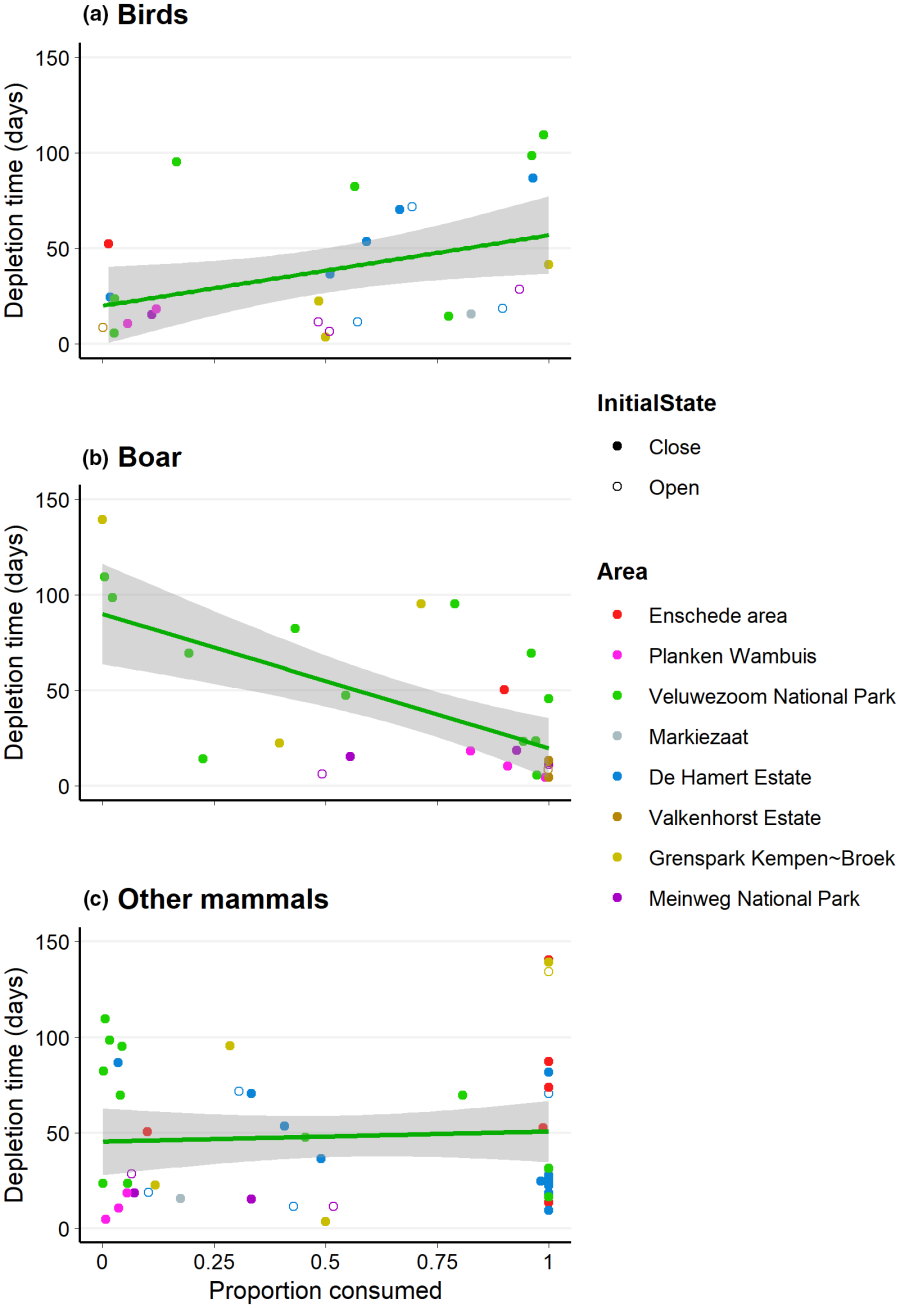
The proportion of carcass consumption attributed to birds (a), boar (b), or other mammals (c) versus the days until carcass depletion.

We found a longer time to carcass depletion when a larger proportion was consumed by birds (Figure 6a; *β* = 40.897, SE = 13.459, df = 22.418, *p* = .013). When boar consumed a larger proportion, the time to depletion was shorter (Figure 6b; *β* = −65.706, SE = 18.163, df = 22.566, *p* = .001). We found no effect of proportion consumed by other mammals on the time to depletion (Figure 6c; *β* = −3.509, SE = 13.876, df = 43.614, *p* = .802). None of the covariates – mean daily temperature (birds: *β* = 2.060, SE = 1.265, df = 13.316, *p* = .127; boar: *β* = 0.147, SE = 1.242, df = 21.002, *p* = .907; other mammals: *β* = 0.973, SE = 1.090, df = 33.620, *p* = .378) or carcass initial state (birds: *β* = −19.262, SE = 13.145, df = 19.072, *p* = .159; boar: *β* = −23.791, SE = 20.716, df = 19.138, *p* = .265; other mammals: *β* = 0.039, SE = 13.726, df = 41.816, *p* = .998) – was significant for any of the scavenger groups.

## 4 DISCUSSION

In this study, we aimed to examine how tree cover and carcass detection by facultative avian and mammalian scavengers, in areas without vultures and without top predators, influenced carcass consumption by different scavenger groups, hence time to carcass depletion. In general, we found tree cover not to be the dominant factor determining carcass exploitation by the different scavenger groups. Our results showed, for instance, that mean daily temperature was a better predictor for the time of first detection and first scavenging by birds than tree cover (Table S2.1). The carcass decomposition process is intrinsically linked to temperature‐dependent biochemical processes (e.g. Carter et al., 2007; DeVault et al., 2003; Matuszewski et al., 2010) but the effect of ambient temperature on vertebrate scavenger activity seems contradictory. On the one hand, decreasing ambient temperature has been shown to increase scavenger activity (e.g. Gomo et al., 2020; Selva et al., 2005), while on the other hand, enhanced ambient temperatures might facilitate earlier carcass detection due to increased olfactory cues (e.g. Inagaki et al., 2022; Peers et al., 2020). However, it remains unclear why we only found an effect of ambient temperature on first detection and first scavenging by birds, while we did not find an effect for boars or other mammals.

Despite that first detection or first scavenging by boars was not influenced by tree cover, it took longer before initially opened carcasses were first scavenged by boars than initially closed carcasses, while the other scavenger groups were not influenced by carcass initial state (Table S1.1). Boars are known to change their behaviour to avoid anthropogenic disturbances – in our case manually opened carcasses (e.g. Fradin & Chamaillé‐Jammes, 2023; Johann et al., 2020). Moreover, wild boar is extremely sensitive to olfactory cues (e.g. Lavelle et al., 2017). It remains, however, unclear why birds and other mammals were not influenced by carcass initial state. Common ravens, for example, quickly learn the potential danger of humans (e.g. Blum et al., 2022), and it has been described that red foxes change their daily activity patterns when human disturbance is high (e.g. Díaz‐Ruiz et al., 2016).

We assumed that birds would exploit carcasses more in open habitats due to their use of eye‐sight (e.g. Ruxton & Houston, 2004; Selva et al., 2005). However, common ravens – the most abundant bird species during our study, contributing to 70 per cent of the bird observations (Table S1.1) – can locate carcasses even in densely forested areas (Rösner et al., 2005). In boreal forests, ravens can even become forest specialists, breeding and foraging inside large natural forests (Andren, 1992). It has also been described how ravens build their nests in forest edges, from where they can reach both open and forested areas, although they would slightly prefer to forage in open habitats (e.g. Dunk et al., 1997). Common buzzards – with 21 per cent of observations the second most abundant bird species in our study (Table S1.1) – strongly prefer to forage in rugged areas (e.g. Sergio et al., 2005) or open habitats like meadows (e.g. Kitowski, 2000; Wikar et al., 2008; Wuczyński, 2005). Therefore, it does not seem evident that the scavenging birds in our study were driven by tree cover, as has been described for vultures (e.g. Arrondo et al., 2019; Oliva‐Vidal et al., 2022). Although we can only speculate about this, it might be that facultative avian scavengers rely on vultures for locating carcasses and respond to tree cover only when vultures are present.

Mammalian scavengers would be mostly driven by olfactory cues when detecting carcasses (e.g. Ruxton & Houston, 2004; Selva et al., 2005; Stahler et al., 2002), which does not automatically mean that they would sooner detect carcasses under denser tree cover. Red fox – with 66 per cent of the observations the most abundant mammal in our study (Table S1.1) – generally prefers cover‐rich habitats but might shift towards more open areas when proved to be beneficial (e.g. Lucherini et al., 1995; White et al., 2006). Pine martens are predominantly active in forested areas and even avoid open habitats, while beech martens use both open and forested areas, even visiting man‐made objects and inhabiting buildings (e.g. Goszczyński et al., 2007). Boars would mostly forage in open habitats close to forest edges, enabling them to escape into the forest in case of danger (e.g. Geisser & Reyer, 2004; Meriggi & Sacchi, 2001). Moreover, in general, mammals are typically more vulnerable to predation in open areas compared to birds, but when large carnivores are absent, the overall mammalian willingness to scavenge in open areas might increase (e.g. Allen et al., 2015), which might have contributed to our findings. In addition, the bird species in our study are mostly diurnal (e.g. Butet et al., 2010; Loretto et al., 2016), while the mammals are mostly nocturnal or crepuscular (e.g. Díaz‐Ruiz et al., 2016; Keuling et al., 2008). We recommend studying this in more detail in future studies since we were not able to analyse this in our study due to technical limitations.

We found that a larger proportion of carcasses was consumed by birds when birds sooner detected them for the first time (Figure 4a) and by boars when boars sooner scavenged for the first time (Figure 4b). Birds are more active in the early stages of decomposition (Wenting et al., 2022). Corvids – common ravens and carrion crow in our study – are known to forage in large flocks (e.g. Marzlufi & Heinrich, 1991; Rösner et al., 2005), although larger flocks do not necessarily represent larger feeding rates (Marzlufi & Heinrich, 1991). Boars are known for their social behaviour and tend to scavenge in large groups (e.g. Dardaillon, 1988; Maselli et al., 2014). These aspects might, however, have caused some unintended bias due to the method we used to calculate the proportion of carcass consumed per scavenger group. The number of observations was multiplied by the number of individuals, meaning that the number of observations of birds and boar might be overestimated compared to the observations of other mammals. The other mammals did not typically forage in large groups, were generally more active during the later stages of decomposition, and detected carcasses later compared to birds and boars (Wenting et al., 2022), which might explain why time to first detection or first scavenging by other mammals did not affect the proportion of carcass consumed by other mammals (Figure 4c).

In general, mammals – both boar and other mammals in our study – have larger bite sizes than birds (e.g. Van Gils et al., 2007). This might explain why the time to carcass depletion was not influenced by time to first detection or first scavenging by birds (Figure 5a), and that a larger proportion of carcass consumed by birds even resulted in a longer depletion time (Figure 6a). Thus, when boars or other mammals detected or scavenged from carcasses for the first time, this might have had a larger effect on carcass depletion time, as our results suggest, both for boars (Figure 5b + Figure 6b) and other mammals (Figure 5c). It remains unknown, however, why a larger proportion of carcass consumed by other mammals did not speed up the time to depletion (Figure 6c).

In conclusion, our results showed that tree cover may not be the dominant factor driving carcass exploitation by facultative vertebrate scavengers in areas without obligate scavengers or large predators. Carcasses decomposed faster when they were sooner detected or scavenged for the first time by boars or other mammals, and when boars consumed higher proportions of the carcasses. This is in line with idea that wild boar plays a key role in areas without obligate scavengers (Wenting et al., 2022), although their behaviour might be less predictable compared to vultures. Wild boar' presence does not automatically result in faster carcass decomposition, but carcass consumption by wild boar does. As a result, we speculate that scavenging by wild boar might have a larger accelerating effect on nutrient cycles compared to other vertebrate facultative scavengers. Thus, when obligate scavengers or large predators are absent, carcass exploitation by facultative scavengers, particularly wild boar, determines the carcass decomposition process, which is not related to a habitat characteristic like tree cover.

## AUTHOR CONTRIBUTIONS

**Elke Wenting:** Conceptualization (lead); data curation (lead); formal analysis (lead); funding acquisition (equal); investigation (lead); methodology (equal); project administration (lead); resources (lead); supervision (equal); validation (lead); visualization (lead); writing – original draft (lead), writing ‐ review & editing (lead). **Patrick A. Jansen:** Conceptualization (supporting); funding acquisition (equal); methodology (supporting); writing – original draft (supporting). **Luke Pattipeilohy:** Conceptualization (supporting); formal analysis (supporting); investigation (supporting); writing – original draft (supporting). **Peter van Lunteren:** Conceptualization (supporting); formal analysis (supporting); investigation (supporting); writing – original draft (supporting). **Henk Siepel:** Conceptualization (supporting); methodology (supporting); supervision (equal); writing – original draft (supporting). **Frank van Langevelde:** Conceptualization (supporting); funding acquisition (equal); methodology (supporting); supervision (equal); writing – original draft (supporting).

## CONFLICT OF INTEREST STATEMENT

No actual or potential conflicts of interest are declared by the authors.

## Supporting information


Appendix S1

Appendix S2

## Data Availability

The complete dataset used in this study, including details about the monitored carcasses, is available through Figshare: https://doi.org/10.6084/m9.figshare.23634000.
