# Supplementary material for: Influence of tree cover on carcass detection and consumption by facultative vertebrate scavengers
Source: Ecol Evol. 2024 Apr 1;14(4):e10935. doi: 10.1002/ece3.10935 (PMC10985364; doi:10.1002/ece3.10935)
Supplement: Supplementary file 1 — Appendix S1 [file ECE3-14-e10935-s002.docx]

Table S1 Number of observations per area of facultative vertebrate scavenger species, belonging to the functional scavenger groups birds, boar, or other mammals.

| Area | Birds | | | Boar | Other mammals | | | | | | |
| --- | --- | --- | --- | --- | --- | --- | --- | --- | --- | --- | --- |
|  | B. buteo | C. corax | C. corone | S. scrofa | C. lupus familias | F. catus | M. foina | M. martes | M. meles | M. putorius | V. vulpes |
| Enschede area | 5 |  |  | 9 |  |  | 130 |  | 2 | 392 | 41 |
| Planken Wambuis | 119 | 11 |  | 1670 |  |  |  |  |  |  | 279 |
| Veluwezoom National Park | 1006 | 4390 | 16 | 3970 | 1 |  |  | 52 | 34 |  | 421 |
| Markiezaat | 27 |  | 479 |  |  |  |  |  |  |  | 122 |
| De Hamert Estate | 235 | 1163 | 11 |  | 50 | 5 | 47 |  | 9 | 117 | 821 |
| Valkenhorst Estate | 3 |  |  | 1076 | 2 |  |  |  |  |  |  |
| Grenspark Kempen~Broek | 91 |  | 224 | 285 | 2 | 56 | 26 |  |  | 1 | 58 |
| Meinweg National Park | 194 |  |  | 344 |  |  | 8 |  |  | 1 | 88 |
| *Total observations* | *1680* | *5564* | *730* | *7291* | *55* | *61* | *211* | *52* | *45* | *511* | *1830* |
| *% of scavenger group* | *21%* | *70%* | *9%* | *100%* | *2%* | *2%* | *8%* | *2%* | *1.5%* | *18.5%* | *66%* |
