# Supplementary material for: Influence of tree cover on carcass detection and consumption by facultative vertebrate scavengers
Source: Ecol Evol. 2024 Apr 1;14(4):e10935. doi: 10.1002/ece3.10935 (PMC10985364; doi:10.1002/ece3.10935)
Supplement: Supplementary file 2 — Appendix S2 [file ECE3-14-e10935-s001.docx]

Overview of the test statistics.

Table S2.1 Test statistics belonging to prediction 1: the denser the tree cover, the longer it takes before carcasses are first detected and first scavenged by birds, but the faster carcasses are first detected and first scavenged by boars or other mammals.

| Mixed effects Cox model  (fixed factors) | Coefficient (β) | SE | z | p |
| --- | --- | --- | --- | --- |
| Birds - first detection, tree cover |  |  |  |  |
| *- Tree cover* | -0.837 | 0.584 | -1.43 | 0.150 |
| *- Temperature* | -0.117 | 0.044 | -2.69 | 0.007* |
| *- Carcass initial state* | 0.349 | 0.414 | 0.84 | 0.400 |
| Birds - first scavenging, tree cover |  |  |  |  |
| *- Tree cover* | -0.684 | 0.619 | -1.11 | 0.270 |
| *- Temperature* | -0.153 | 0.051 | -3.02 | 0.003* |
| *- Carcass initial state* | 0.317 | 0.459 | 0.69 | 0.490 |
| Boar - first detection, tree cover |  |  |  |  |
| *- Tree cover* | 1.292 | 0.663 | 1.95 | 0.052 |
| *- Temperature* | -0.096 | 0.050 | -1.94 | 0.053 |
| *- Carcass initial state* | -1.078 | 0.667 | -1.62 | 0.110 |
| Boar - first scavenging, tree cover |  |  |  |  |
| *- Tree cover* | 0.126 | 0.644 | 0.20 | 0.840 |
| *- Temperature* | -0.060 | 0.050 | -1.20 | 0.230 |
| *- Carcass initial state* | -1.555 | 0.776 | -2.00 | 0.045* |
| Other mammals - first detection, tree cover |  |  |  |  |
| *- Tree cover* | 0.913 | 0.560 | 1.63 | 0.100 |
| *- Temperature* | -0.035 | 0.042 | -0.85 | 0.400 |
| *- Carcass initial state* | 0.238 | 0.429 | 0.55 | 0.580 |
| Other mammals - first scavenging, tree cover |  |  |  |  |
| *- Tree cover* | 0.866 | 0.601 | 1.44 | 0.150 |
| *- Temperature* | 0.040 | 0.048 | 0.83 | 0.410 |
| *- Carcass initial state* | -0.423 | 0.484 | -0.87 | 0.380 |

| Mixed effects Cox model  (random effects) | Std. dev. | Variance |  | Std. dev. | Variance |
| --- | --- | --- | --- | --- | --- |
| Birds - first detection, tree cover |  |  | - first scavenging, tree cover |  |  |
| *- Area* | 0.017 | <0.001 |  | 0.020 | <0.001 |
| *- Carcass species* | 0.384 | 0.148 |  | 0.348 | 0.121 |
| *- Start month* | 0.017 | <0.001 |  | 0.020 | <0.001 |
| Boar - first detection, tree cover |  |  | - first scavenging, tree cover |  |  |
| *- Area* | 1.115 | 1.244 |  | 1.336 | 1.786 |
| *- Carcass species* | 0.020 | <0.001 |  | 0.020 | <0.001 |
| *- Start month* | 0.020 | <0.001 |  | 0.020 | <0.001 |
| Other mammals - first detection, tree cover |  |  | - first scavenging, tree cover |  |  |
| *- Area* | 0.171 | 0.029 |  | 0.372 | 0.138 |
| *- Carcass species* | 0.019 | <0.001 |  | 0.823 | 0.677 |
| *- Start month* | 0.692 | 0.479 |  | 0.772 | 0.596 |

Table S2.2 Test statistics belonging to prediction 2: the denser the tree cover, the lower the proportion of carcass consumed by birds, but the higher the proportion consumed by boars or other mammals.

| Generalized linear mixed-effects model (GLMM)  (fixed factors) | Coefficient (β) | SE | z | p |
| --- | --- | --- | --- | --- |
| Birds - proportion consumed, tree cover |  |  |  |  |
| *Intercept* | -0.041 | 0.845 | -0.048 | 0.962 |
| *- Tree cover* | -0.889 | 0.952 | -0.934 | 0.350 |
| *- Temperature* | -0.012 | 0.090 | -0.132 | 0.895 |
| *- Carcass initial state* | -0.147 | 0.658 | -0.224 | 0.823 |
| Boar - proportion consumed, tree cover |  |  |  |  |
| *Intercept* | 0.560 | 0.740 | 0.757 | 0.449 |
| *- Tree cover* | -0.379 | 0.846 | -0.448 | 0.654 |
| *- Temperature* | 0.008 | 0.057 | 0.142 | 0.887 |
| *- Carcass initial state* | 0.437 | 0.995 | 0.439 | 0.661 |
| Other mammals - proportion consumed, tree cover |  |  |  |  |
| *Intercept* | -0.407 | 0.579 | -0.704 | 0.482 |
| *- Tree cover* | 0.337 | 0.637 | 0.529 | 0.597 |
| *- Temperature* | 0.062 | 0.041 | 1.513 | 0.130 |
| *- Carcass initial state* | 0.154 | 0.501 | 0.308 | 0.758 |

| Generalized linear mixed-effects model (GLMM)  (random factors) | Variance (σ^2^) | Std. dev. |
| --- | --- | --- |
| Birds - proportion consumed, tree cover |  |  |
| *- Area* | <0.001 | <0.001 |
| *- Carcass species* | <0.001 | <0.001 |
| *- Start month* | <0.001 | <0.001 |
| Boar - proportion consumed, tree cover |  |  |
| *- Area* | <0.001 | <0.001 |
| *- Carcass species* | <0.001 | <0.001 |
| *- Start month* | <0.001 | <0.001 |
| Other mammals proportion consumed, tree cover |  |  |
| *- Area* | <0.001 | <0.001 |
| *- Carcass species* | <0.001 | <0.001 |
| *- Start month* | <0.001 | <0.001 |

Table S2.3 Test statistics belonging to prediction 3: the sooner carcasses are first detected or first scavenged by birds, boars or other mammals, the higher the proportion of carcass consumed by these groups.

| Linear mixed-effects model (LMM)  (fixed factors) | Coefficient (β) | SE | z | p |
| --- | --- | --- | --- | --- |
| Birds - first detection, proportion consumed |  |  |  |  |
| *Intercept* | 0.261 | 0.638 | 0.409 | 0.683 |
| *- Days first detection* | -0.125 | 0.037 | -3.360 | <0.001* |
| *- Temperature* | 0.005 | 0.066 | 0.078 | 0.938 |
| *- Carcass initial state* | 0.027 | 0.569 | 0.047 | 0.962 |
| Birds - first scavenging, proportion consumed |  |  |  |  |
| *Intercept* | 0.084 | 0.742 | 0.122 | 0.910 |
| *- Days first scavenging* | -0.068 | 0.041 | -1.655 | 0.098 |
| *- Temperature* | 0.059 | 0.071 | 0.820 | 0.412 |
| *- Carcass initial state* | -0.159 | 0.668 | -0.238 | 0.812 |
| Boar - first detection, proportion consumed |  |  |  |  |
| *Intercept* | 0.463 | 0.713 | 0.650 | 0.516 |
| *- Days first detection* | 0.005 | 0.023 | 0.220 | 0.826 |
| *- Temperature* | -0.006 | 0.054 | -0.112 | 0.910 |
| *- Carcass initial state* | 0.433 | 1.011 | 0.428 | 0.668 |
| Boar - first scavenging, proportion consumed |  |  |  |  |
| *Intercept* | 0.717 | 0.699 | 1.026 | 0.305 |
| *- Days first scavenging* | -0.038 | 0.012 | -3.176 | 0.001* |
| *- Temperature* | 0.093 | 0.053 | 1.749 | 0.080 |
| *- Carcass initial state* | 0.874 | 0.789 | 1.107 | 0.268 |
| Other mammals - first detection, proportion consumed |  |  |  |  |
| *Intercept* | -0.312 | 0.504 | -0.618 | 0.537 |
| *- Days first detection* | 0.008 | 0.016 | 0.489 | 0.625 |
| *- Temperature* | 0.066 | 0.040 | 1.663 | 0.100 |
| *- Carcass initial state* | 0.075 | 0.475 | 0.159 | 0.874 |
| Other mammals - first scavenging, proportion consumed |  |  |  |  |
| *Intercept* | -0.176 | 0.611 | -0.288 | 0.774 |
| *- Days first scavenging* | 0.002 | 0.011 | 0.202 | 0.840 |
| *- Temperature* | 0.073 | 0.044 | 1.660 | 0.100 |
| *- Carcass initial state* | -0.106 | 0.516 | -0.206 | 0.837 |

*Cont. Table S2.3*

| Linear mixed-effects model (LMM)  (random factors) | Variance (σ^2^) | Std. dev. |  | Variance (σ^2^) | Std. dev. |
| --- | --- | --- | --- | --- | --- |
| Birds - first detection, proportion consumed |  |  | - first scavenging, proportion consumed |  |  |
| *- Area* | <0.001 | <0.001 |  | <0.001 | <0.001 |
| *- Carcass species* | <0.001 | <0.001 |  | <0.001 | <0.001 |
| *- Start month* | <0.001 | <0.001 |  | <0.001 | <0.001 |
| Boar - first detection, proportion consumed |  |  | - first scavenging, proportion consumed |  |  |
| *- Area* | <0.001 | <0.001 |  | <0.001 | <0.001 |
| *- Carcass species* | <0.001 | <0.001 |  | <0.001 | <0.001 |
| *- Start month* | <0.001 | <0.001 |  | <0.001 | <0.001 |
| Other mammals - first detection, proportion consumed |  |  | - first scavenging, proportion consumed |  |  |
| *- Area* | <0.001 | <0.001 |  | <0.001 | <0.001 |
| *- Carcass species* | <0.001 | <0.001 |  | <0.001 | <0.001 |
| *- Start month* | <0.001 | <0.001 |  | <0.001 | <0.001 |

Table S2.4 Test statistics belonging to prediction 4: the carcass decomposition speed is not influenced by time to first detection or first scavenging by birds, but is accelerated by time to first detection or first scavenging by boars or other mammals.

| Generalized linear mixed-effects model (GLMM)  (fixed factors) | Coefficient (β) | SE | df | t | p |
| --- | --- | --- | --- | --- | --- |
| Birds - depletion, first detection |  |  |  |  |  |
| *Intercept* | 25.671 | 14.715 | 14.572 | 1.745 | 0.102 |
| *- Days first detection* | 1.079 | 0.908 | 27.871 | 1.188 | 0.245 |
| *- Temperature* | 2.814 | 1.558 | 21.378 | 1.807 | 0.085 |
| *- Carcass initial state* | -27.709 | 14.706 | 26.118 | -1.884 | 0.071 |
| Birds - depletion, first scavenging |  |  |  |  |  |
| *Intercept* | 35.238 | 16.186 | 13.452 | 2.177 | 0.048 |
| *- Days first scavenging* | -0.900 | 1.059 | 27.793 | -0.850 | 0.403 |
| *- Temperature* | 3.089 | 1.542 | 19.716 | 2.003 | 0.059 |
| *- Carcass initial state* | -27.946 | 14.981 | 24.716 | -1.865 | 0.074 |
| Boar - depletion, first detection |  |  |  |  |  |
| *Intercept* | 32.410 | 18.003 | 11.985 | 1.800 | 0.097 |
| *- Days first detection* | 1.130 | 0.409 | 12.975 | 2.765 | 0.016* |
| *- Temperature* | -0.478 | 1.325 | 13.114 | -0.360 | 0.724 |
| *- Carcass initial state* | -8.307 | 17.753 | 24.324 | -0.468 | 0.644 |
| Boar - depletion, first scavenging |  |  |  |  |  |
| *Intercept* | 35.708 | 13.107 | 14.924 | 2.724 | 0.016 |
| *- Days first scavenging* | 1.230 | 0.190 | 23.867 | 6.466 | <0.001* |
| *- Temperature* | -1.444 | 0.952 | 25.421 | -1.516 | 0.142 |
| *- Carcass initial state* | -11.289 | 13.843 | 25.945 | -0.815 | 0.422 |
| Other mammals - depletion, first detection |  |  |  |  |  |
| *Intercept* | 25.068 | 11.974 | 22.766 | 2.093 | 0.048 |
| *- Days first detection* | 1.231 | 0.364 | 45.649 | 3.379 | 0.002* |
| *- Temperature* | 0.716 | 0.944 | 36.434 | 0.759 | 0.453 |
| *- Carcass initial state* | -0.869 | 11.016 | 46.855 | -0.079 | 0.938 |
| Other mammals - depletion, first scavenging |  |  |  |  |  |
| *Intercept* | 14.993 | 10.509 | 13.974 | 1.427 | 0.176 |
| *- Days first scavenging* | 1.304 | 0.228 | 44.993 | 5.731 | <0.001* |
| *- Temperature* | 1.174 | 0.780 | 33.161 | 1.468 | 0.151 |
| *- Carcass initial state* | -3.036 | 9.433 | 46.822 | -0.322 | 0.749 |

*Cont. Table S2.4*

| Generalized mixed-effects model (GLMM)  (random factors) | Variance (σ^2^) | Std. dev. |  | Variance (σ^2^) | Std. dev. |
| --- | --- | --- | --- | --- | --- |
| Birds - depletion, first detection |  |  | - depletion, first scavenging |  |  |
| *- Area* | 0.0 | 0.00 |  | 0.0 | 0.00 |
| *- Carcass species* | 0.0 | 0.00 |  | 0.0 | 0.00 |
| *- Start month* | 111.5 | 10.56 |  | 101.5 | 10.07 |
| Boar - depletion, first detection |  |  | - depletion, first scavenging |  |  |
| *- Area* | 353.6 | 18.80 |  | <0.001 | 0.002 |
| *- Carcass species* | 0.0 | 0.00 |  | <0.001 | 11.85 |
| *- Start month* | 257.4 | 16.04 |  | <0.001 | <0.001 |
| Other mammals - depletion, first detection |  |  | - depletion, first scavenging |  |  |
| *- Area* | 153.9 | 12.40 |  | 104.1 | 10.202 |
| *- Carcass species* | 0.0 | 0.00 |  | 11.46 | 3.386 |
| *- Start month* | 183.4 | 13.54 |  | 87.62 | 9.361 |

Table S2.5 Test statistics belonging to prediction 5: the carcass decomposition speed is not influenced by the proportion of carcass consumed by birds, but is accelerated when the proportions consumed by boars or other mammals increase.

| Generalized linear mixed-effects model (GLMM) | Coefficient (β) | SE | df | t | p |
| --- | --- | --- | --- | --- | --- |
| Birds - depletion, proportion consumed |  |  |  |  |  |
| *Intercept* | 6.902 | 13.459 | 16.930 | 0.513 | 0.615 |
| *- Proportion consumed* | 40.897 | 15.241 | 22.418 | 2.683 | 0.013* |
| *- Temperature* | 2.060 | 1.265 | 13.316 | 1.629 | 0.127 |
| *- Carcass initial state* | -19.262 | 13.145 | 19.072 | -1.465 | 0.159 |
| Boar - depletion, proportion consumed |  |  |  |  |  |
| *Intercept* | 87.818 | 17.063 | 10.744 | 5.147 | <0.001* |
| *- Proportion consumed* | -65.706 | 18.163 | 22.566 | -3.618 | 0.001* |
| *- Temperature* | 0.147 | 1.242 | 21.002 | 0.118 | 0.907 |
| *- Carcass initial state* | -23.791 | 20.719 | 19.138 | -1.148 | 0.265 |
| Other mammals - depletion, proportion consumed |  |  |  |  |  |
| *Intercept* | 37.674 | 16.168 | 17.615 | 2.330 | 0.032 |
| *- Proportion consumed* | -3.509 | 13.876 | 43.614 | -0.253 | 0.802 |
| *- Temperature* | 0.973 | 1.090 | 33.620 | 0.893 | 0.378 |
| *- Carcass initial state* | 0.039 | 13.726 | 41.816 | 0.003 | 0.998 |

| Generalized linear mixed-effects model (GLMM)  (random factors) | Variance (σ^2^) | Std. dev. |
| --- | --- | --- |
| Birds - depletion, proportion consumed |  |  |
| *- Area* | 95.126 | 9.753 |
| *- Carcass species* | <0.001 | <0.001 |
| *- Start month* | 4.357 | 2.087 |
| Boar - depletion, proportion consumed |  |  |
| *- Area* | <0.001 | 5.386 |
| *- Carcass species* | <0.001 | <0.001 |
| *- Start month* | <0.001 | 0.002 |
| Other mammals - depletion, proportion consumed |  |  |
| *- Area* | 503.8 | 22.45 |
| *- Carcass species* | <0.001 | <0.001 |
| *- Start month* | 321.7 | 17.94 |
